# Handbook of General Therapeutics

**Published:** 1885-11

**Authors:** 


### Book Reviews.

Handbook of General Therapeutics, in seven volumes. By Professor H. Von Ziemssen. Vols. I., II., III. 8vo. New York: William Wood & Co. 1885.

Vol. I. Contains an introduction to the work by Von Ziemssen, in which the editor justly states that the late brilliant advances in the domain of therapeutics are due to the employment of the scientific method of inquiry, and that the work before us is but a careful collation and elaboration of widely distributed material having an important bearing upon the subject of general therapeutics. This material, being the result of close scientific analysis, must afford a solid foundation for the structure it is to support. This volume, entitled "Dietary of the Sick," treats of the analysis and value of various food stuffs, animal and vegetable; also of the stimulants, alcohol, tea, coffee, etc., presenting valuable tables of their chemical analysis. This is followed by a chapter on digestion and utilization of nutriment and the demand of the organism for nutriment. Under the heading of "Special Dietetics for the Sick," the author gives some scientific information regarding the administration and effect of food in febrile and anomalous states of the general nutrition and tissue change.

The book proper closes with a chapter on dietetic methods of treatment; *i. e.*, vegetarianism, grape cure, milk cure, etc.

As an appendix, a short discussion of the value of koumiss cures is added.

Much still remains to be learned regarding the action of foods in metabolism under various pathological conditions. The same may be said regarding the effects of the varied morbid processes upon metabolism, and the quantity and quality of the food necessary to meet the special requirements of the economy imposed by these processes. The results thus far obtained and offered in this volume are strictly in accord with the latest physiological inquiry and chemical analysis, and their practical application, given in some degree, will be of value to the practitioner.

VOL. II. *Methods of Treatment.*—The first four chapters are devoted to the antipyretic methods of treatment, embracing a discussion upon the nature and efficacy of the various applications of moist cold, and the internal administration of antipyretic drugs in febrile affections, especially with a view of reducing high temperature. The cold bath, in different forms, is the popular method of treatment in fevers, especialiy typhoid, among the Germans, and their results are certainly satisfactory. Liebermeister gives statistics which show how the mortality in typhoid fever was reduced from 27 per cent., when symptomatic treatment was employed, to 9 per cent. when complete antipyretic measures were used.

Next follows an interesting and practical dissertation on the antiphlogistic methods of treatment, including blood-letting and transfusion, their effects on the system, also giving the results of experimental research. As regards blood-letting in disease, the writer properly remarks that no definite rule can be laid down, and hopes that the waste of blood is forever gone.

The volume closes with a description of the cutaneous administration of medicine—the methods, appliances and various drugs used. A full list of drugs is given, together with their doses and special effects upon the system.

VOL. III. *Respiratory Therapeutics.*—Part first discusses the subject of the inhalation of remedies in the form of vapors in the treatment of diseases of the respiratory tract. Special action of inhalations, necessary apparatus, and a long list of medicinal agents adapted to this method are given, together with the treatment of special maladies. Part second deals with the treatment of diseases of the respiratory and circulatory organs by alterations of air pressure. The various appliances, the effects of their use upon the system, and the special indications for their employment, are set forth *in extenso.*

This book is comprehensive in its style and covers a subject as important as it is obscure and unsatisfactory. It supplies a want and will be read with interest and profit by the practicing physician. Written by a man of unusual experience in the treatment of this class of diseases, the work can be received with confidence.

